# Arf GTPase-activating proteins SMAP1 and AGFG2 regulate the size of Weibel-Palade bodies and exocytosis of von Willebrand factor

**DOI:** 10.1242/bio.058789

**Published:** 2021-09-01

**Authors:** Asano Watanabe, Hikari Hataida, Naoya Inoue, Kosuke Kamon, Keigo Baba, Kuniaki Sasaki, Rika Kimura, Honoka Sasaki, Yuka Eura, Wei-Fen Ni, Yuji Shibasaki, Satoshi Waguri, Koichi Kokame, Yoko Shiba

**Affiliations:** 1 Faculty of Science and Engineering, Iwate University, Morioka, 020-8551, Japan; 2 Department of Molecular Pathogenesis, National Cerebral and Cardiovascular Center, Osaka, 564-8565, Japan; 3 Department of Biotechnology, National Kaohsiung Normal University, Kaohsiung, 80201, Taiwan; 4 Department of Anatomy and Histology, Fukushima Medical University, Fukushima, 960-1295, Japan

**Keywords:** Arf, ArfGAP, Secretory granule, Exocytosis, VWF, WPB

## Abstract

Arf GTPase-Activating proteins (ArfGAPs) mediate the hydrolysis of GTP bound to ADP-ribosylation factors (Arfs), which are critical to form transport intermediates. ArfGAPs have been thought to be negative regulators of Arfs; however, accumulating evidence indicates that ArfGAPs are important for cargo sorting and promote membrane traffic. Weibel-Palade bodies (WPBs) are cigar-shaped secretory granules in endothelial cells that contain von Willebrand factor (vWF) as their main cargo. WPB biogenesis at the Golgi was reported to be regulated by Arf and their regulators, but the role of ArfGAPs has been unknown. In this study, we performed siRNA screening of ArfGAPs to investigate the role of ArfGAPs in the biogenesis of WPBs. We found two ArfGAPs, SMAP1 and AGFG2, to be involved in WPB size and vWF exocytosis, respectively. SMAP1 depletion resulted in small-sized WPBs, and the lysosomal inhibitor leupeptin recovered the size of WPBs. The results indicate that SMAP1 functions in preventing the degradation of cigar-shaped WPBs. On the other hand, AGFG2 downregulation resulted in the inhibition of vWF secretion upon Phorbol 12-myristate 13-acetate (PMA) or histamine stimulation, suggesting that AGFG2 plays a role in vWF exocytosis. Our study revealed unexpected roles of ArfGAPs in vWF transport.

## Abstract (precis)

**Summary:** The Arf GTPase-activating proteins SMAP1 and AGFG2 regulate the size of Weibel-Palade bodies and exocytosis of von Willebrand factor.

## INTRODUCTION

Arf GTPase-Activating proteins (ArfGAPs) mediate hydrolysis of GTP bound to ADP-ribosylation factors (Arfs), small GTP binding proteins critical to the formation of transport vesicles (Kahn et al., 2008; Sztul et al., 2019; Tan and Gleeson, 2019). As ArfGAPs ‘inactivate’ Arf-GTP by GTP hydrolysis, it had been thought that ArfGAPs were terminators of Arfs, however, ArfGAP1, the first identified and most well-studied ArfGAP, were reported to play an important role in cargo sorting during the formation of COPI vesicles (Pepperkok et al., 2000; Nickel et al., 1998; Lanoix et al., 2001; Yang et al., 2002; Liu et al., 2005; Shiba et al., 2011). Our previous studies revealed that ArfGAP1 promotes the hydrolysis of Arf-GTP in the presence of coatomer and its specific cargos, to promote the polymerization of coatomer (Luo et al., 2009; Shiba et al., 2011). These reports indicate that ArfGAP1 plays a critical role in cargo sorting through its GAP activity during COPI vesicle formation (Spang et al., 2010; East and Kahn, 2011; Kahn, 2011; Hsu, 2011; Beck et al., 2011; Shiba and Randazzo, 2012). There are 31 genes of ArfGAPs in human (Shiba and Randazzo, 2012) and other ArfGAPs could also play roles in promoting Arf-dependent transport (Lewis et al., 2004; Dai et al., 2004; Shiba et al., 2010, 2013), however, the way in which ArfGAPs function in the sorting of secretory protein remains poorly understood.

In endothelial cells, von Willebrand factor (vWF) is synthesized in the endoplasmic reticulum (ER), transported to the Golgi apparatus, and then packaged into secretory granules called Weibel-Palade bodies (WPBs). Upon stimulation, WPBs fuse with the plasma membrane (PM) and vWF is released into blood vessels to recruit platelets for blood clotting. WPBs have characteristic cigar-shaped structures containing vWF as the main cargo. AP-1 is an Arf-dependent clathrin adaptor that mediates binding between clathrin and its cargo. Depletion of an AP-1 subunit results in the inhibition of biogenesis of WPBs from the *trans*-Golgi network (TGN) (Lui-Roberts et al., 2005). It was suggested that AP-1 and clathrin play roles in sorting vWF at the Golgi. However, other reports have suggested that AP-1 plays a role in the removal of lysosomal proteins from immature secretory granules (ISGs) in pancreatic β-cells, parotid acinar cells and neuroendocrine cells (Klumperman et al., 1998; Kakhlon et al., 2006), rather than the formation of ISGs. Therefore, the way in which AP-1 functions in both TGN and ISGs to sort each cargo remains unknown.

In this study, whether ArfGAPs regulate the sorting of vWF, we searched for ArfGAPs that regulate the formation of WPBs. First, we performed siRNA screening of ArfGAPs for WPB morphology using HEK293 cells, which have a high efficiency of transfection. Then, we used human umbilical vein endothelial cells (HUVECs) that have endogenous WPBs for a second screening. We identified two ArfGAPs, SMAP1 and AGFG2, which regulated WPB size and vWF exocytosis, respectively. Our study revealed the unexpected function of ArfGAPs in vWF transport.

## RESULTS

### siRNA-screening of ArfGAPs in HEK293 and HUVECs

Our previous studies showed that depletion, but not overexpression, of ArfGAPs inhibits intracellular transport (Shiba et al., 2013, 2010, 2011). We therefore used the same approach to inhibit ArfGAP expression using siRNAs. There are 31 genes encoding ArfGAPs in humans. Of these, AGAP4 siRNA is able to target AGAP4 to AGAP10 mRNAs; we used a total of 25 siRNAs against ArfGAPs (Shiba et al., 2013). For siRNA screening, we first used HEK293 cells, because HEK293 cells have a high efficiency of transfection, and the overexpression of the vWF gene in HEK293 is known to produce pseudo-WPBs (Romani de Wit et al., 2003; Michaux et al., 2003). We transfected the siRNAs of 25 ArfGAPs together with the GFP-vWF plasmid into the HEK293 cells and used confocal microscopy to examine whether the cigar-shaped structure of pseudo-WPBs was altered. We eliminated the cells that have apparently cigar-shaped structure of pseudo-WPBs (see Materials and Methods). Fourteen siRNAs (ArfGAP3, SMAP1, GIT2, AGFG2, ADAP1, 2, ASAP2, 3, ACAP1, 3, ARAP3, AGAP3, 4, 11) remained in first screening and these were subjected for second screening. In the second screening, six siRNAs (SMAP1, GIT2, AGFG2, ASAP2, ACAP3, AGAP11) among 14 siRNAs remained and these were subjected for subsequent analysis (Fig. S1A). We transfected with these six siRNAs and quantified the phenotype. We classified the cells based upon the structure of their GFP-vWF (Fig. S1B). Class I cells had cigar-shaped GFP-vWF, class II had small puncta of GFP-vWF, and class III had no apparent structure. We analyzed more than 30 cells per siRNA, and calculated the proportion of cells, which fell into each class. These six siRNA transfected cells showed a decrease in class I cells, and increases in class II and III cells, compared with control siRNA-transfected cells (Fig. S1C).

Next, we tested whether SMAP1, GIT2, AGFG2, ASAP2 ACAP3 and AGAP11 siRNAs can affect the endogenous WPB structure in HUVECs. We electroporated these six siRNAs into HUVECs, stained them with anti-vWF antibody, and processed them for immunofluorescence. We classified the cells according to their WPB structure (Fig. S1D). Class I had cigar-shaped WPBs, class II had small puncta of WPBs, class III had no apparent structure, and class IV had no expression of vWF. We classified more than 40 cells in an experiment, and repeated the experiment three times. As shown in Fig. S1E, the cells transfected with SMAP1 and AGFG2 siRNAs showed a decrease in class I cells compared with that in control cells (control 51%; SMAP1 21.4%; AGFG2 21.5%, *P*<0.05 for both). These results suggest that SMAP1 and AGFG2 siRNAs affect the structure of endogenous WPB in HUVECs.

### SMAP1 depletion leads to small WPBs

**Fig. 1.**
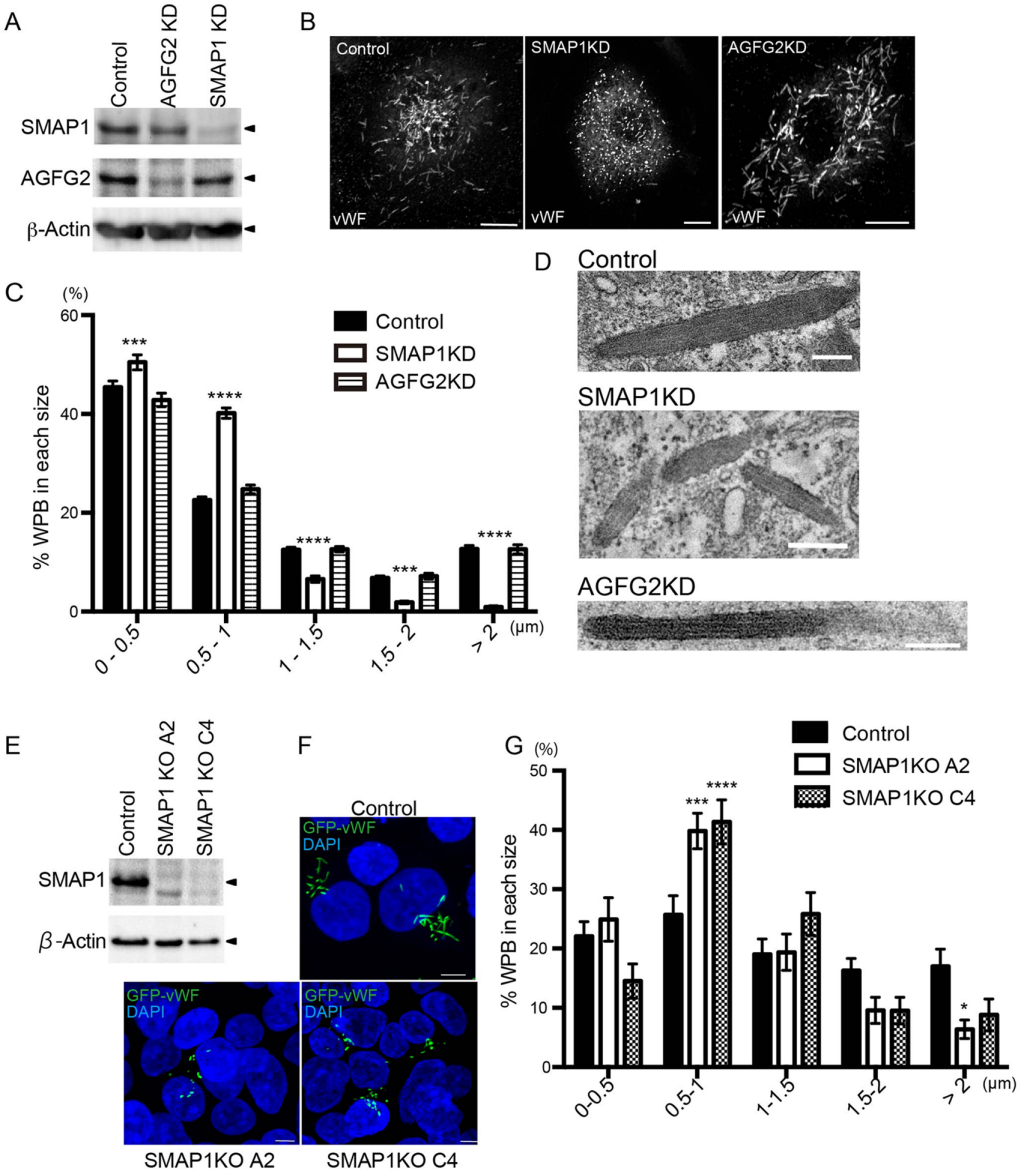
**SMAP1 depletion results in small WPBs.** (A) HUVECs electroporated with control, SMAP1, or AGFG2 siRNA were subjected to immunoblotting with anti-SMAP1, AGFG2, and β-Actin antibodies. (B) HUVECs electroporated with siRNAs of control, SMAP1, or AGFG2, were stained with anti-vWF. Scale bars: 10 μm. (C) The size of WPBs of more than ten cells was measured, and the experiment was repeated three times (*n=*32 for all samples). The percentages of each WPB size are shown in the histogram. Two-way ANOVA followed by Sidak's multiple comparisons test was performed for each siRNA. SMAP1KD cells have more WPBs less than 1 μm, and fewer WPBs larger than 1 µm. AGFG2KD cells do not show any difference from control cells. ***P<0.001, ****P<0.0001, ns; not significant, error bar, s.e.m. (D) TEM images of control, SMAP1KD, and AGFG2KD cells are shown. In SMAP1KD cells, smaller WPBs were detected, while in AGFG2KD cells, cigar-shaped WPBs were observed. Scale bars: 200 nm. (E) Control and SMAP1KO cell lines A2 and C4 derived from HEK293 were subjected to immunoblotting with anti-SMAP1 and anti-β-actin antibodies. (F) Control and SMAP1KO A2 and C4 cells were transfected with GFP-vWF. Control cells have cigar-shaped pseudo-WPBs, while SMAP1 KO cells show small pseudo-WPBs. Scale bar: 5 μm. (G) The experiment was repeated three times, and the size of pseudo-WPBs analyzed in more than 30 cells (Control, *n=*42, A2; *n=*39, C4; *n=*40). Two-way ANOVA followed by Sidak's multiple comparisons test was performed. SMAP1KO cell lines have smaller WPBs. *P<0.05, ***P<0.001,****P<0.0001, error bar, s.e.m.

To confirm the downregulation of SMAP1 and AGFG2 proteins, we performed immunoblotting using anti-SMAP1 and anti-AGFG2 antibodies for HUVECs electroporated with SMAP1 and AGFG2 siRNAs (Fig. 1A). We observed that SMAP1 and AGFG2 were downregulated by more than 70% and 80%, respectively. Hereafter, we refer to these SMAP1 and AGFG2 siRNA-transfected cells as SMAP1 knockdown (KD), and AGFG2KD cells, respectively. To quantify the changes in morphology of WPBs in SMAP1KD and AGFG2KD cells, we analyzed the size of the WPBs in immunofluorescence images (Fig. 1B,C). As shown in Fig. 1C, in SMAP1KD cells, the proportion of WPBs of length 0–0.5 µm and 0.5–1 µm were increased (0–0.5 µm: control 45.4%; SMAP1KD 50.5%; *P*<0.001; 0.5–1 µm: control 22.5%; SMAP1KD 40.2%; *P*<0.0001). WPBs of length 0.5–1 µm had a twofold increase in SMAP1KD cells. On the other hand, in SMAP1KD cells, the number of WPBs larger than 1 µm was decreased (1–1.5 µm: control 12.5%; SMAP1KD 6.6%; *P*<0.001; 1.5–2 µm: control 6.8%; SMAP1KD 1.9%, *P*<0.0001; >2 µm: control 12.7%; SMAP1KD 0.9%; *P*<0.0001). These results indicate that the WPBs were smaller in SMAP1KD cells. We performed the same analysis in AGFG2KD cells. In the screening, we found AGFG2 to form small WPBs (Fig. S1D). However, when we incubated primary antibody longer than 2 h, we could see cigar-shaped WPBs in AGFG2KD cells (Fig. 1B). As a result, we found no significant difference in WPB size (Fig. 1C). In AGFG2KD cells, vWF could be packaged more tightly and less accessible to anti-vWF antibody (see Discussion).

We further examined the structure of WPBs using transmission electron microscopy (TEM). As shown in Fig. 1D, smaller WPBs were observed in SMAP1KD cells, whereas cigar-shaped WPBs were found in control and AGFG2KD cells.

To examine the off-target effects of SMAP1 siRNA, we established two cell lines of SMAP1 knockout (KO) by HEK293 cells. We did not use HUVECs, to avoid using the cells after passage 5. We confirmed complete depletion of SMAP1 protein by western blotting in A2 and C4 cell lines (Fig. 1E). We transfected GFP-vWF into the control, A2 and C4 cell lines, and analyzed the size of the WPBs (Fig. 1F,G). As in SMAP1KD cells, in A2 and C4 cell lines, WPBs sized between 0.5–1.0 µm were increased (control 24.5%; A2 39.2%; *P*<0.001; C4 41.6%, *P*<0.0001) and WPBs >2 µm in length were decreased in the A2 cell line (control 16.5%; A2 5.6%, *P*<0.05). The results confirmed that SMAP1 depletion causes smaller WPBs.

### vWF secretion was inhibited in AGFG2KD cells

**Fig. 2.**
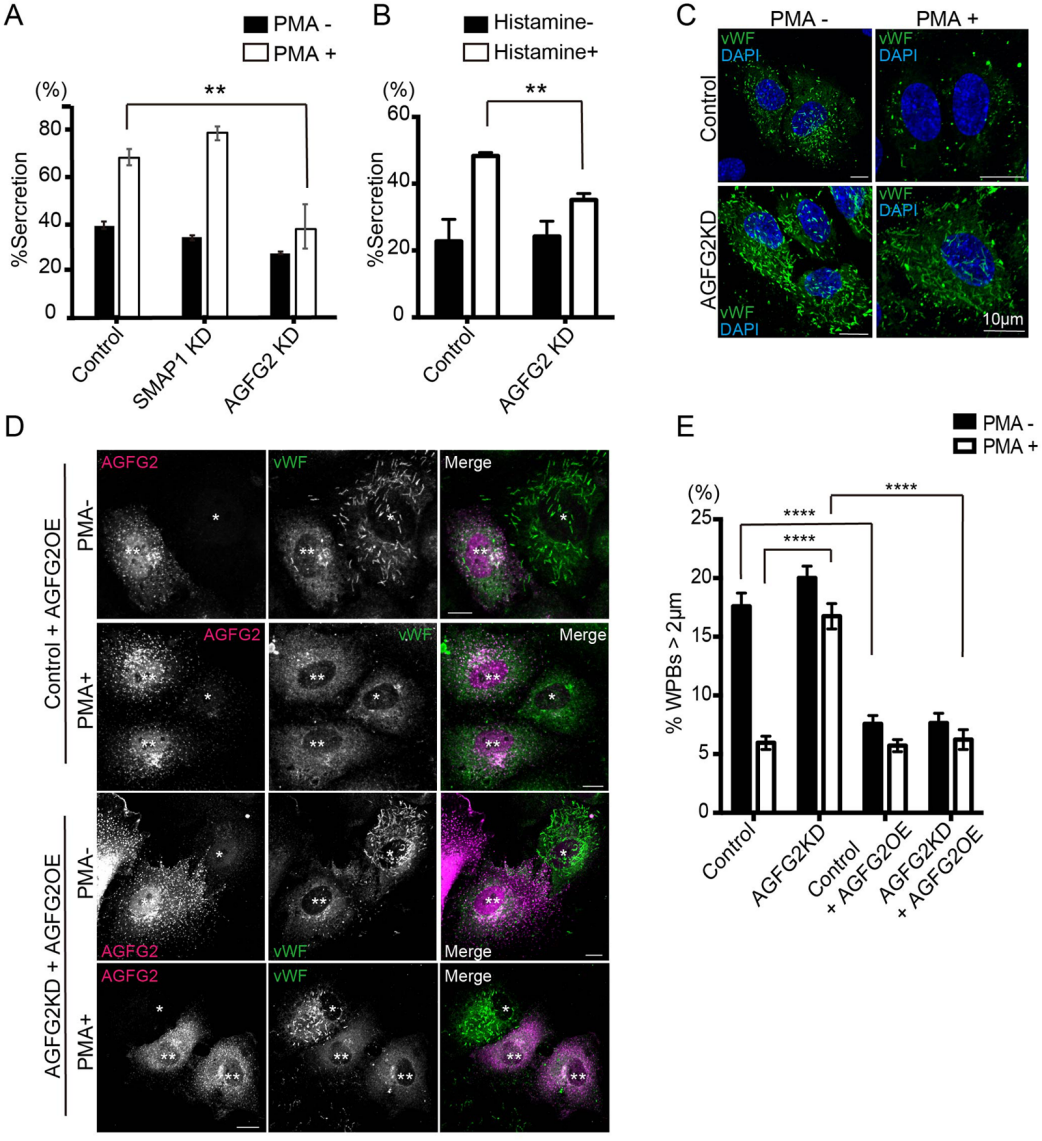
**AGFG2 plays a role in vWF secretion.** (A) HUVECs electroporated with control, SMAP1, and AGFG2 siRNAs were stimulated with DMSO or 100 ng/ml PMA for 30 min, the medium and the lysates were collected, and the vWF amount was quantified using ELISA. The percentage vWF was calculated as the vWF in the medium divided by the total vWF. The experiments were repeated three times (*n=*3). Two-way ANOVA with Dunnett's multiple comparison test was performed with or without PMA. PMA-stimulated vWF secretion was significantly decreased in AGFG2KD cells. **P<0.01, error bar, s.e.m. (B) The same analysis was performed by 100 μM histamine. Histamine-stimulated vWF secretion was significantly decreased in AGFG2KD cells. **P<0.01. (C) HUVECs electroporated with control and AGFG2 siRNAs were stimulated with DMSO or 100 ng/ml PMA for 30 min, and stained with anti-vWF (green) and DAPI (blue). Following PMA treatment, control cells lost cigar-shaped WPBs, whereas AGFG2KD cells retained cigar-shaped WPBs. Scale bars: 10 μm. (D) HUVECs were electroporated with control or AGFG2 siRNAs, together with AGFG2 siRNA-resistant plasmids. The cells were treated with PMA for 30 min, and then stained for anti-vWF (green) and anti-AGFG2 (magenta) antibodies. AGFG2 overexpressing (OE) cells are indicated by **, and the cells without overexpression of AGFG2 are indicated by *. AGFG2OE cells lost cigar-shaped WPBs with or without PMA in both control and AGFG2KD cells. Scale bars: 10 μm. (E) WPB size was analyzed for 15 cells per experiment, and the experiment was repeated three times (*n=*45 for all samples). WPBs >2 μm are shown. Two-way ANOVA with Sidak's multiple comparison test was performed. ****P<0.0001, error bar, s.e.m.

To address the role of SMAP1 and AGFG2 in vWF secretion, we performed enzyme-linked immunosorbent assay (ELISA). We used Phorbol 13-myristate 12-acetate (PMA) as a secretagogue because it is highly efficient for stimulation (Zografou et al., 2012). We stimulated cells using 100 ng/ml PMA for 30 min, quantified the amount of vWF in the medium and the lysates, and calculated the percentage of secretion as the amount vWF in the medium divided by the total vWF in the medium and lysates. As shown in Fig. 2A, in control cells PMA stimulation increased vWF secretion around twofold compared with PMA-unstimulated cells (control PMA- 40.7%; PMA+74.5%). In SMAP1KD cells, the secretion of vWF with or without PMA showed no significant difference compared with that in the control cells. In contrast, PMA-stimulated secretion was halved in AGFG2 KD cells (control 74.5%; AGFG2KD 37.6%, *P*<0.01). For PMA-unstimulated secretion, we observed some decrease in secretion in AGFG2KD cells, but the difference was not significant (control 40.7%; AGFG2 27.6%; *P*=0.14). We also performed the secretion assay by histamine (Fig. 2B). In AGFG2KD cells, histamine-stimulated secretion of vWF was decreased (control 48.3%; AGFG2KD 35.1%, *P*<0.01). The secretion without histamine was not significantly changed (control 22.7%; AGFG2KD 24.2%, *P*=0.86). These results suggest that AGFG2 plays an important role in the stimulation-dependent secretion of vWF. We also quantified vWF secretion by immunofluorescence (Fig. 2C–E). In PMA-unstimulated cells, cigar-shaped WPBs were observed in the control cells. In contrast, PMA-stimulated cells have only small puncta, suggesting that most of the cigar-shaped WPBs were secreted upon stimulation. We quantified the size of the WPBs (Fig. S2), and found that following PMA treatment the proportion of WPBs greater than 2 µm in length was decreased (PMA−16%; PMA+5.2%; *P*<0.0001) and the proportion of WPBs between 0.5–1 μm was increased (PMA−30.7%; PMA+38.6%; *P*<0.0001). As the decrease of WPBs >2 µm was marked following PMA treatment, with a decrease of 70%, we focused on quantifying WPBs >2 µm. We performed immunofluorescence in AGFG2KD cells with or without PMA (Fig. 2C). In AGFG2KD cells, many of cigar-shaped WPBs remained, even following PMA treatment. We quantified WPBs >2 µm (Fig. 2E), and found that AGFG2KD cells had around threefold WPBs >2 µm compared with control cells (control PMA+5.9%; AGFG2KD PMA+16.7%; *P*>0.0001). Therefore, the immunofluorescence results were consistent with the ELISA results, supporting the idea that PMA-stimulated vWF secretion was inhibited in AGFG2KD cells. To check for off-target effects of AGFG2 siRNA, we overexpressed the siRNA-resistant AGFG2 gene with AGFG2 siRNA in HUVECs and performed immunofluorescence. As shown in Fig. 2D, AGFG2 overexpressed cells (AGFG2OE) lost cigar-shaped WPBs, even without PMA in control cells, and only cytoplasmic and perinuclear vWF remained. AGFG2 overexpression in AGFG2KD cells also led to the loss of cigar-shaped WPBs with or without PMA. We quantified WPBs >2 µm and found that AGFG2OE cells had decreased WPBs >2 µm in both control and AGFG2KD cells without PMA (Fig. 2E). PMA did not stimulate further vWF secretion. These results indicate that AGFG2 overexpression promotes vWF secretion and overcomes the AGFG2KD phenotype. Our analyses indicated that AGFG2 plays an important role in stimulation-dependent secretion of vWF.

### WPB maturation was not highly perturbed in SMAP1KD and AGFG2KD cells

**Fig. 3.**
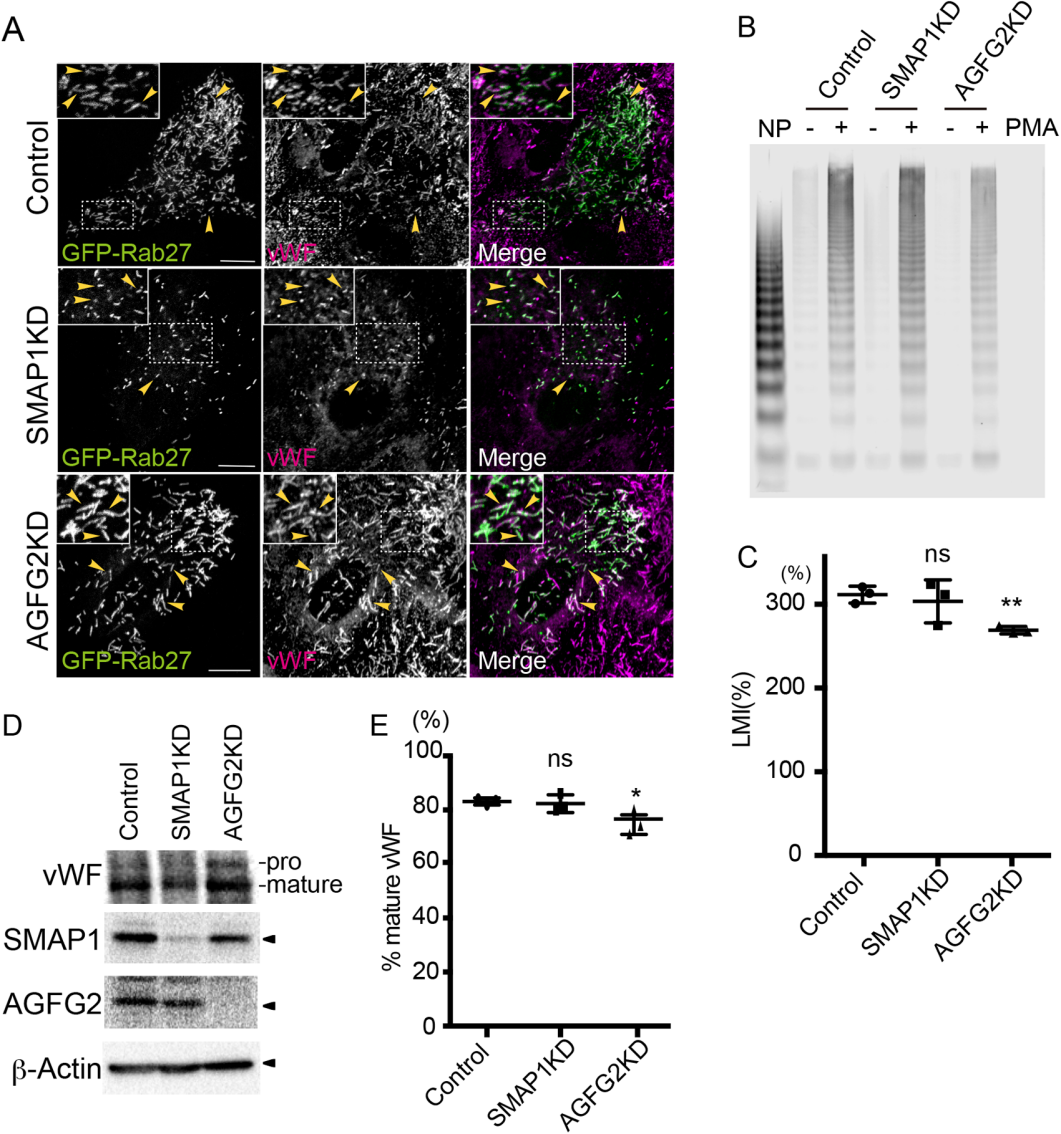
**WPB maturation is not highly perturbed in SMAP1KD and AGFG2KD cells.** (A) HUVECs were electroporated with GFP-Rab27a and siRNAs, as indicated, and incubated for 72 h. The cells were stained with anti-vWF (magenta). Arrowheads show colocalization between GFP-Rab27a and vWF. Small WPBs in SMAP1KD cells also colocalized with GFP-Rab27a. Scale bars: 10 μm. (B) HUVECs electroporated with siRNAs as indicated were incubated for 72 h and treated with 100 ng/ml PMA for 30 min. The medium was collected and the same amount of medium was subjected to agarose gel electrophoresis followed by anti-vWF western blotting. Normal human plasma (NP) was used as control. The smallest bands are considered to be dimers, and multiple bands are observed as multimerization increases. HMW-vWF was decreased in AGFG2KD cells. (C) HMW-vWF was normalized by total multimers per lane, according to the large-multimer ratio. The LMI was calculated as the relative value of the large-multimer ratio to NP. The experiment was repeated three times, and unpaired *t*-tests was performed. AGFG2KD cells secreted less HMW-vWF, ns; not significant, **P<0.01, error bar, s.d. (D) HUVECs electroporated with siRNAs, as previously described, and the cell lysates were subjected to western blotting. Mature vWF was observed in all cells. (E) The experiment in D was repeated three times, and the percentage of mature vWFs was calculated as the ratio to total pro- and mature-vWF. Unpaired *t*-tests were performed. A decrease of ∼9% was observed in AGFG2KD cells, but most of the vWF matured, ns; not significant, *P<0.05, error bar, s.d.

To investigate WPBs maturation in SMAP1KD and AGFG2KD cells, we examined the colocalization of vWF and the secretory granule marker, GFP-Rab27a. Rab27 has been reported to be a late stage marker for WPBs (Hannah et al., 2003). vWF was partially colocalized with GFP-Rab27a in control, SMAP1KD and AGFG2KD cells (Fig. 3A). We quantified the colocalization using Pearson's correlation coefficient (PCC). The PCCs between vWF and GFP-Rab27a in control, SMAP1KD and AGFG2KD cells were 0.1±0.08 (*n*=14), 0.14±0.23 (*n=*10) and 0.26±0.11 (*n=*5), respectively. The values were all positive, confirming that GFP-Rab27a is recruited to WPBs in all cases. In SMAP1KD cells, WPBs were smaller but we did observe the colocalization of GFP-Rab27a with the small puncta of vWF (Fig. 3A). To confirm that the vWF punctate signal does not come from other compartments, such as early endosomes, we stained vWF with the early endosome marker, EEA1 (Fig. S3). vWF did not colocalize with EEA1 in control, SMAP1KD or AGFG2KD cells. The PCCs between vWF and EEA1 in control, SMAP1KD and AGFG2KD cells were −0.18±0.07 (*n=*5), −0.16±0.07 (*n=*5), and −0.02±0.002 (*n=*5), suggesting that vWF was not mis-localized to early endosomes. We also stained vWF with the TGN marker TGN46 in control, SMAP1KD and AGFG2KD cells. Although punctate signal in SMAP1KD cells is apparent, the TGN architecture was not highly changed in SMAP1KD cells (Fig. S3).

To examine whether vWF multimerization was affected, we stimulated HUVECs using PMA, and analyzed the multimerization of vWF in the medium (Fig. 3B). We found that in AGFG2KD cells, high-molecular weight (HMW)-vWF appeared to be decreased compared to that in control or SMAP1KD cells. We calculated the large-multimer ratio as the ratio of HMW-vWF to total secreted vWF, and compared each sample using the large-multimer index (LMI, see Materials and Methods) (Fig. 3C). LMIs in control and SMAP1KD cells were not significantly different (control 311.6%; SMAP1KD 303.5%; *P*>0.05), whereas AGFG2KD cells had lower HMW-vWF (control 311.6%; AGFG2KD 269.1% *P*<0.01). These results suggest that the amount of HMW-vWF in the medium of AGFG2KD cells was decreased but not in SMAP1KD cells. As the PMA-stimulated secretion of vWF was inhibited in AGFG2KD cells (Fig. 2A), lower levels of HMW-vWF in AGFG2KD cells could come from the inhibition of fusion of cigar-shaped WPBs, rather than inhibition of vWF multimerization in cells. We analyzed vWF processing in lysates by a protease furin that recycles between the TGN and the cell surface (Wagner et al., 1986; Wise et al., 1990; Molloy et al., 1994). We observed cleaved vWF in SMAP1KD and AGFG2KD cells as well as in control cells (Fig. 3D). We quantified the percentage of cleaved vWF per total cleaved and non-cleaved vWF (Fig. 3E). Although we found that AGFG2KD cells had an approximately 9% decrease of cleaved vWF (control 83.2%; AGFG2KD 74.5%; *P*<0.05), but most of vWF were cleaved in AGFG2KD cells.

Overall, these results indicate that vWF transport to WPBs is not highly perturbed in SMAP1KD and AGFG2KD cells.

### Leupeptin recovered the size of WPBs in SMAP1-depleted cells

**Fig. 4.**
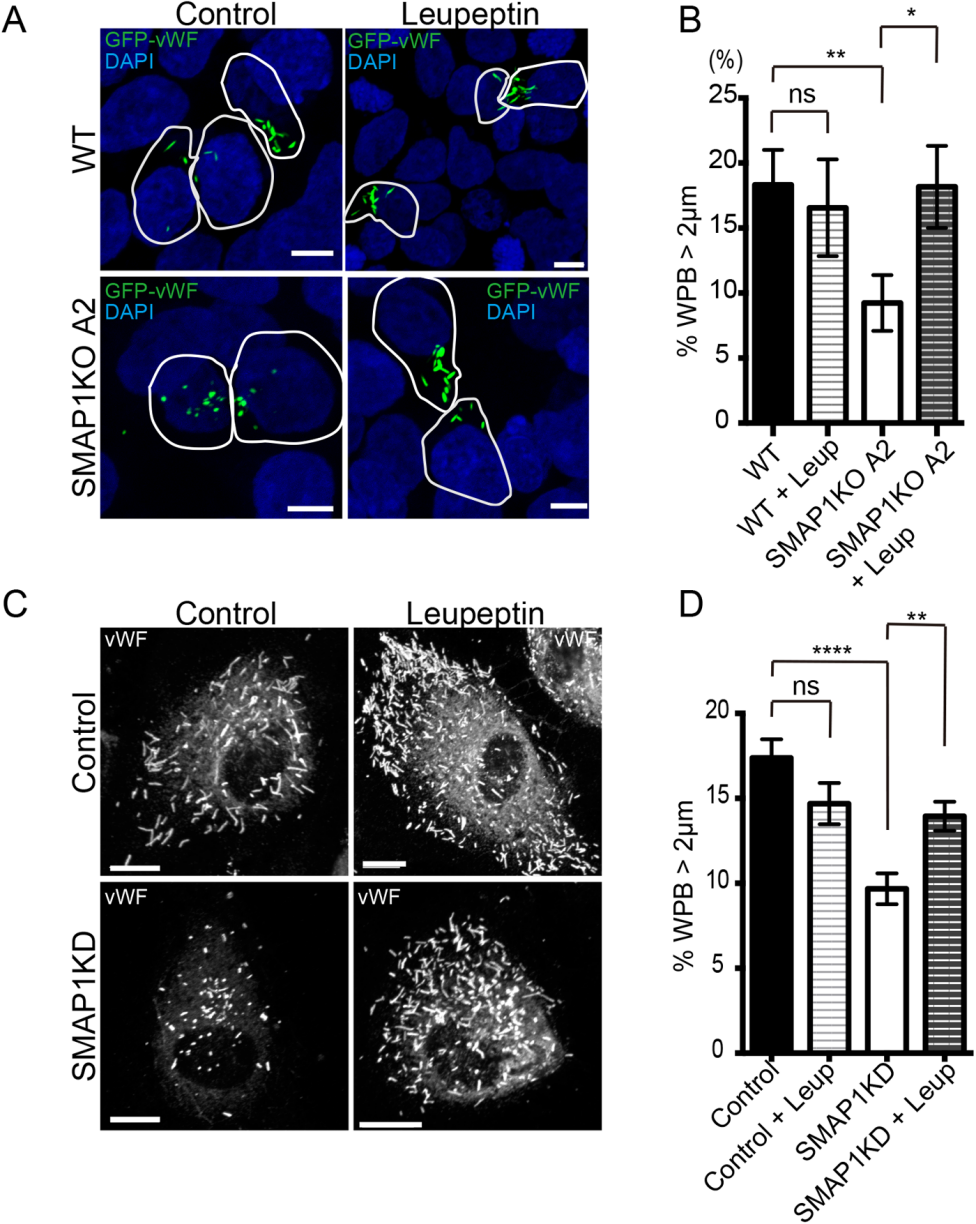
**Leupeptin restored the size of WPBs in SMAP1-depleted cells.** (A) Wild-type HEK293 and SMAP1KO A2 cell lines were transfected with GFP-vWF, and 40 h post-transfection, the cells were treated with 1 mg/ml Leupeptin for 8 h. The cells were fixed and processed for immunofluorescence. SMAP1KO cells had small pseudo-WPBs, but leupeptin treatment recovered long pseudo-WPBs. Scale bars: 5 μm. (B) Pseudo-WPB size was analyzed for more than seven cells per experiment, and the experiment was repeated three times (WT; *n=*35, WT+Leup; *n=*25, SMAP1KO; *n=*30, SMAP1KO+Leup; *n=*39). Pseudo-WPBs >2 μm are shown. Mann–Whitney tests were performed. The size of pseudo-WPBs was recovered by leupeptin in SMAP1KO cells. ns; not significant, *P<0.05, **P<0.01, error bar, s.e.m. (C) HUVECs were electroporated with control or SMAP1 siRNAs, and 60 h post-transfection, 1 mg/ml leupeptin was added, and the cells incubated for 12 h. The cells were fixed and processed for immunofluorescence. Cigar-shaped WPBs were recovered with leupeptin in SMAP1KD cells. Scale bars: 10 μm. (D) WPB size was analyzed for more than ten cells per experiment and the experiment was repeated three times (Control *n=*36, Control+Leup *n=*33, SMAP1KD; *n*=39, SMAP1KD+Leup; *n*=34). WPBs >2 μm was shown. Mann–Whitney test was performed. WPB size was recovered by Leupeptin treatment in SMAP1KD cells. ns; not significant, **P<0.01, ****P<0.0001, error bar, s.e.m.

So far, our results indicated that SMAP1 plays a role in maintaining WPB size. However, it was not clear how SMAP1 regulates WPB size independent of vWF multimerization/processing or mis-localization of vWF. We investigated whether SMAP1 regulates the degradation of cigar-shaped WPBs. We transfected GFP-vWF into the SMAP1KO cell line A2, and treated them with leupeptin, a soluble lysosome inhibitor that inhibits a wide range of proteases, including cysteine, serine and threonine proteases. We found that in leupeptin-treated cells, cigar-shaped WPBs re-emerged in SMAP1KO cells (Fig. 4A). We quantified the size of the pseudo-WPBs (Fig. 4B) and found that the numbers of WPBs >2 μm was recovered by leupeptin (SMAP1KO 8.2%; SMAP1KO+Leup 17.8%; *P*<0.01). To confirm these results for endogenous WPBs, we used HUVECs electroporated by either control or SMAP1 siRNA. We found that cigar-shaped WPBs were also restored with leupeptin treatment in HUVECs (Fig. 4C). Our quantification confirmed that WPBs >2 μm was recovered by leupeptin (SMAP1KD 9.7%; SMAP1KD+Leup 13.9%; *P*<0.01) (Fig. 4D). These results suggested that SMAP1 depletion accelerated the degradation of cigar-shaped WPBs, so only globular WPBs remained in SMAP1-depleted cells.

## DISCUSSION

In this study, we identified SMAP1 and AGFG2 as ArfGAPs that regulate vWF transport. SMAP1 regulates the size of WPB, probably by inhibiting WPB degradation in the lysosome, and AGFG2 regulates stimulation-dependent secretion of vWF.

Because AP-1 was proposed to be important for vWF sorting in the TGN (Lui-Roberts et al., 2005), we expected to find ArfGAPs involved in vWF sorting. We found that SMAP1KD cells has smaller WPBs and investigated the possibility if SMAP1 regulates vWF localization, multimerization, and processing. However, we could not find clear evidence that SMAP1 regulates these processes (Fig. 3; Fig. S3). Instead, we found that treatment with leupeptin restored the size of WPBs in SMAP1-depleted cells (Fig. 4). Therefore, we assume that SMAP1 plays a role in preventing cigar-shaped WPBs from degradation in lysosomes, and depletion of SMAP1 leads to rapid degradation of cigar-shaped WPBs. WPBs are thought to be degraded by autophagy (Torisu et al., 2013; Wu et al., 2019), although free form of vWF has also been seen in lysosomes (Torisu et al., 2013). Large sized organelles or pathogens could be preferably degraded by autophagy, but physiologically important organelles such as WPBs would escape from autophagy. The way in which SMAP1 prevents WPBs from degradation, is an interesting research question in the future. Arf1, Arf4 and GBF1, a guanine-nucleotide exchange factor (GEF) for Arfs, were reported to be important for vWF transport, but the phenotypes induced by the depletion of these factors were different to that of AP-1 depletion. Considering these results, in conjunction with our own, we hypothesize that AP-1 is important to maintain the structure of the TGN, independent of the Arf-ArfGAP system.

SMAP1 is known to be the GAP for Arf6 (Tanabe et al., 2005). To investigate whether the GAP activity of SMAP1 is important or not, we overexpressed SMAP1 wild type and [R61Q], a GAP-dead mutant of SMAP1 to HUVECs and HEK293 cells. However, the overexpression of wild type and SMAP1[R61Q] both affected the Golgi architecture detected by TGN46, and vWF was accumulated in the ER (data not shown). Therefore, it was difficult to investigate the events after the TGN. Arf6 depletion is reported to affect only slightly on WPBs formation (Lopes-da-Silva et al., 2019) and secretion (Biesemann et al., 2017). An Arf6-GAP, ACAP2 overexpression was reported to inhibit vWF exocytosis but by ACAP2 depletion by its siRNA, the difference was marginal (Biesemann et al., 2017). Consistent with their results, we also did not see the inhibition of WPBs formation in ACAP2 siRNA transfected cells (Fig. S1A). SMAP1 has clathrin binding-domain (Tanabe et al., 2005) and may have function independent of its GAP activity. Which domain of SMAP1 is required for vWF transport, will be investigated in the future work.

We also tried to examine if endogenous SMAP1 colocalizes with vWF in WPBs by immunofluorescence (data not shown). We have not detected significant localization of SMAP1 on WPBs so far. To determine the precise localization of SMAP1, we may need to develop the method by which SMAP1 is tagged in its genome and investigated the SMAP1 localization in its endogenous level of the expression by live-cell imaging without permeabilization. Although Arf had been known to be very cytosolic, live-imaging of Arf using this method succeeded to detect Arf localization in transport intermediates (Bottanelli et al., 2017). These experiments would also help to investigate the phenotypes of SMAP1 mutants.

The SMAP1 gene locus has been identified to be susceptible for pediatric venous thromboembolism (VTE) (Rühle et al., 2017). Physiologically, SMAP1 dysfunction could promote blood clotting. However, our results implicate that SMAP1 depletion causes small WPBs, which would lead to the secretion of less adhesive vWF to platelets, as small WPBs has been reported to secret vWF with lower adhesive activity to platelets (Michaux et al., 2006; Ferraro et al., 2016). Therefore, our results predict that SMAP1 dysfunction would cause bleeding disorder, but physiologically, the effects could be opposite. SMAP1 is expressed in other cell types including hematopoietic lineages (Kobayashi et al., 2014; Kon et al., 2013). SMAP1-deficient mice were once made and exhibited the erythroid hyperplasia and decrease of platelets (Kon et al., 2013). The effects of SMAP1-deficnecy in hematological homeostasis, and relationship to VTE should be addressed in broader context in the future.

We also found AGFG2 plays an important role in vWF exocytosis. Initially, we identified AGFG2 as affecting the morphology of WPBs (Fig. S1). At that time, our protocol uses 90 min incubation of anti-vWF antibody. However, we noticed that when we incubated the anti-vWF antibody more than 2 h, we could detect cigar-shaped WPBs in AGFG2KD cells (Fig. 1B,C). We also detected cigar-shaped WPBs in TEM (Fig. 1D). Subsequent analyses showed WPBs in AGFG2KD cells remained in cells even under PMA (Fig. 2C,D). As less packed vWF within WPBs leads to brighter WPBs probably by higher accessibility of the epitope to anti-vWF (Lopes da Silva et al., 2016), vWF within WPBs that remained longer in AGFG2KD cells would be more packed and less accessible to anti-vWF antibody compared with control cells. That would lead to appear less WPBs in AGFG2KD cells during the screening.

In AGFG2KD cells, secretion of vWF under PMA or histamine is inhibited (Fig. 2A,B). Consistent with this, in multimer analysis, we detected a decrease of HMW-vWF in AGFG2KD cells under PMA treatment (Fig. 3B). Our results suggest that AGFG2 plays an important role in stimulation-dependent secretion of vWF. We also detected the overexpression of AGFG2 stimulated WPB release with or without PMA (Fig. 2D). If AGFG2 plays a role in the fusion of WPBs without PMA as basal secretion, we could have seen the inhibition of unstimulated secretion in AGFG2KD cells, but we could not (Fig. 2A,B). As unstimulated secretion is thought to be the sum of constitutive and basal secretion (Lopes da Silva and Cutler, 2016), further analysis is required for the possibility that AGFG2 regulates basal secretion.

AGFG proteins were reported to lose important amino acids in ArfGAP domain (Schlacht et al., 2013), but human AGFG2 conserves ArfGAP consensus sequence CX2CX16CX2CX4R in ArfGAP domain. To determine whether the GAP activity is required for AGFG2 function, we overexpressed AGFG2 wild type and [R75Q], a GAP-dead mutant of AGFG2 to HUVECs. AGFG2[R75Q] expression also induced secretion of vWF and we have not detected the specific effects of AGFG2[R75Q] expression compared with that of wild-type AGFG2 so far (data not shown). AGFG2 also has FG repeat domain in its C-terminus. Previous studies showed that AGFG2 enhances the activity of HIV Rev for nuclear export of viral mRNAs (Doria et al., 1999). FG repeats are typical for nucleoporins that consist of nuclear pore complex, and known to play an important role in cargo selection in nuclear transport. Whether FG repeat or ArfGAP domain of AGFG2 is important for exocytosis, should be addressed in the future. According to the NCBI Gene database, AGFG2 is highly expressed in salivary gland (Fagerberg et al., 2014). AGFG2 could play a role in exocytosis in other secretory cells.

In this study, we identified SMAP1 and AGFG2 for regulating vWF transport. We revealed the novel roles of SMAP1 in regulating the size of WPBs and of AGFG2 in the vWF exocytosis. Future work will reveal the novel mechanisms of the regulation of organelle size by SMAP1 and exocytosis by AGFG2.

## MATERIALS AND METHODS

### Reagents

ON-TARGET plus non-targeting siRNA #4, siGENOME SMART pool of human SMAP1, GIT2, AGFG2, ASAP2, ACAP3, AGAP11, and human AGFG2 gene were purchased from Horizon Discovery Ltd (Cambridge, UK). GFP-tagged human vWF plasmid (Romani de Wit et al., 2003) was kindly provided by Jan Voorberg (Stichting Sanquin Bloedvoorziening, Netherland) under Materials Transfer Agreement. AGFG2 was inserted into pcDNA3.1 using EcoRI and XbaI without any tag. For siRNA-resistant plasmids, AGFG2 was mutated in four locations with the primer, mutation #1; 5′-TAG TAT TTT TAC AAT CCC GTG GAA ATG AG-3′, 5′-ATT GTA AAA ATA CTA CTT CAG GCT CAG T-3′, #2; 5′-GGT TTG TAG AAA GAT TTG GTT GGG TCT G-3′, 5′-ATC TTT CTA CAA ACC TCA TTT CCA CGG GA-3′, #3; 5′-GAC AAG CCT CGT ACC AGA TTC CAG GGA T-3′, 5′-GGT ACG AGG CTT GTC CGA GCA TCA AAC AG-3′, #4; 5′-AGG AAG CGC GAA GTT GGG GCA GAG GCC A-3′, 5-AAC TTC GCG CTT CCT AAG TCC CCG AAG GA-3 using Prime STAR mutagenesis kit (Takara Bio Inc. Shiga, Japan). GFP-Rab27a was purchased from RikenBRC (Tsukuba, Japan) (Kuroda et al., 2002; Fukuda and Kuroda, 2002; Tsuboi and Fukuda, 2006). Sheep polyclonal anti-vWF antibody (AHP062T) was purchased from Bio-Rad (Hercules, CA, USA), anti-HA (HA.C5) from Abcam (Cambridge, UK), anti-EEA1(3C10) from MBL (Nagoya, Japan), anti-β-actin (8H10D10) from Cell Signaling Technology (Danvers, MA, USA), anti-HA (12CA5) from Thermo Fisher Scientific (Waltham, MA, USA), rabbit polyclonal anti-SMAP1(A114714) and anti-AGFG2 (R08625) from ATLAS antibodies (Stockholm, Sweden), and anti-TGN46 (ab50595) from Abcam. Secondary antibodies of donkey anti-sheep, mouse and rabbit IgG conjugated with Alexa Fluor 488, 568 or 594 and Dylight were purchased from Thermo Fisher Scientific, AffinPure donkey anti-mouse and rabbit IgG conjugated with peroxidase from Jackson ImmunoResearch (West Grove, PA, USA).

### Cell culture

HEK293 cells were maintained by DMEM (Thermo Fisher Scientific) supplemented with 10% Fetal Bovine Serum (Cytiva, Marlborough, MA, USA) and Antibiotic-Antimycotic Stock Solution (NACALAI TESQUE, Inc., Kyoto, Japan). For replating cells, Trypsin-EDTA solution (Merck, Darmstadt, Germany) was used. HUVECs were purchased from Kurabo industries (Osaka, Japan) and Takara. HUVECs were maintained in Endothelial Cell Basal Medium 2 supplemented with Endothelial Cell Growth Medium kits (C22211, C22111, Takara). For replating cells, the cells were washed once with Hepes-buffered saline (Hepes-BSS), trypsinized with Trypsin-EDTA (CC5012, Lonza, Basal, Switzerland) and neutralized with Hepes-BSS/10% FBS. The cells were centrifuged to eliminate Hepes-BSS/10% FBS and plated with the growth medium. The cells were used by passage 5.

### siRNA screening of ArfGAPs

The custom cherry-pick siRNA library was constructed from 25 human ArfGAPs and control siRNA by Horizon Discovery Ltd. 15×10^4^ HEK 293 cells were seeded on collagen-coated coverslips (Cellmatrix, Type IV, Nitta gelatin, Osaka, Japan) and transfected with 5 nM siRNA using RNAiMax (Thermo Fisher Scientific) using a reverse-transfection protocol. The knockdown efficiency was confirmed by ArfGAP3 siRNA using western blotting, and almost 100% knockdown was achieved. After 24 h of siRNA transfection, the medium was changed and the cells were transfected with GFP-vWF using Lipofectamine 3000, as per the manufacturer's protocol (Thermo Fisher Scientific). The medium was changed after 24 h and incubated for another 24 h. The siRNA transfection period was 72 h. The cells were fixed with 4% PFA/PBS for 15 min, quenched using 50 mM NH_4_Cl/PBS for more than 20 min, stained with DAPI (Merck) for 2 min, and mounted with Mowiol. A confocal microscope (Nikon C2, Tokyo, Japan) was used for capturing images using the 60× objective (NA1.40) and the 100× objective (NA 1.45). We visualized all samples using the same setting of the confocal microscope, and eliminated cells that had apparently cigar-shaped pseudo-WPBs with no changes compared with those in control cells. We kept the cells for which we could not immediately judge whether there were any changes of pseudo-WPBs or not, such as there were only few cells on coverslips or only few cells expressing GFP-vWF, for next analysis. Fourteen siRNAs (ArfGAP3, SMAP1, GIT2, AGFG2, ADAP1, 2, ASAP2, 3, ACAP1, 3, ARAP3, AGAP3, 4, 11) were kept in the first screening (Fig. S1A). We transfected these 14 siRNAs again into HEK293 cells, and analyzed them using the same protocol as the first screening. We eliminated the cells that have apparently cigar-shaped pseudo-WPBs. Six siRNAs (SMAP1, GIT2, AGFG2, ASAP2, ACAP3, AGAP11) were kept in the second screening. For these six siRNAs, the images of four fields that formed a single larger square were captured. More than 30 cells were analyzed (Fig. S1B,C).

### Immunofluorescence

Between 10–30×10^4^ HUVECs were electroporated with 50 pmol of control or ArfGAP siRNAs using the Neon transfection system (Thermo Fisher Scientific) at 1350 V for 30 ms, seeded on gelatin-coated (Merck) coverslips. After 16–24 h, the medium was changed. We noticed that the medium change promoted vWF secretion, therefore we incubated the cells for 48 h after the medium change, to allow the cells to form new WPBs. Seventy-two hours post-transfection with siRNAs, the cells were washed in PBS, fixed in 4% PFA/PBS for 15 min, quenched using 50 mM NH_4_Cl/PBS for more than 20 min, permeabilized with 0.2% Triton X-100/PBS for 10 min and incubated with blocking solution (5% BSA/PBS) for more than 30 min. The cells were incubated with primary antibodies diluted in 0.02% Triton X-100/1% BSA/PBS for more than 90 min, washed with PBS for 5 min three times, and incubated with the secondary antibody diluted in 0.02% Triton X-100/1% BSA/PBS for 45 min. The cells were washed with PBS for 5 min three times, stained with DAPI for 3 min, washed, and mounted in Mowiol. The images were captured using a confocal microscope.

For Phorbol 12-myristate 13-acetate (PMA, Merck) treatment, HUVECs were electroporated and 48 h after the medium was changed, the cells were serum starved for 1 h, and then treated with 100 ng/ml PMA for 30 min, as described for ELISA. The cells were fixed and processed for immunofluorescence.

For leupeptin treatment, 15×10^4^ wild-type HEK293 cells or SMAP1 KO A2 cells were transfected with GFP-vWF with Lipofectamine LTX (Thermo Fisher Scientific) by reverse transfection, as per the manufacturer's protocol. After 16–20 h, the medium was changed. Twenty hours after medium change, leupeptin (Peptide Institute Inc. Osaka, Japan) was added to the medium to produce a final concentration of 1 mg/ml, and the cells were incubated for 8 h. The cells were washed twice, fixed with 4% PFA/PBS, quenched with 50 mM NH_4_Cl/PBS, stained with DAPI and mounted with Mowiol. For HUVECs, 20×10^4^ HUVECs were electroporated with control or SMAP1 siRNA. For HUVECs, 20×10^4^ HUVECs were electroporated with control or SMAP1 siRNA. After 24 h, the medium was changed. Thirty-six hours after the medium change, leupeptin was added to the medium to a final concentration of 1 mg/ml, and the cells were incubated for 12 h. The cells were fixed and processed for immunofluorescence.

### Image analysis and statistics

Images of control, SMAP1KD, and AGFG2KD cells were captured using confocal microscopy. For quantification, the same settings were used in the control, SMAP1KD and AGFG2KD cells. To measure the size of WPBs, the confocal images were projected at maximum projection, and the WPB size was measured using the Fiji software (Schindelin et al., 2012). The ‘Analyze Particles’ plugin was used to calculate Feret's diameter as the length of WPBs. More than ten cells per coverslip were analyzed and the experiment was repeated more than three times. More than 30 cells were quantified in total. A histogram was created using bins of 0.5 µm, as follows: 0–0.5=<0, 0.499>, 0.5–1=<0.5, 0.999>, 1–1.5=<1–1.499>, 1.5–2=<1.5–1.999>, 2<=<2, infinity>. For PCC, a 3D mask was created using Fiji, and colocalization was quantified using the Coloc 2 plugin. The value was described with±s.d. Statistical analyses were performed using GraphPad PRISM version 6 software (GraphPad Software, San Diego, CA, USA, www.graphpad.com).

### Western blotting

180×10^4^ HUVECs were electroporated and seeded on a six-well plate. After 24 h, the medium was changed and the cells incubated for 48 h. The cells were lysed in 100 µl of lysis buffer (50 mM Tris-HCl, pH 7.5, 100 mM NaCl, 2 mM MgCl_2_, 1 mM DTT, 1% Triton X-100) with protease inhibitor cocktail (NACALAI) for 30 min on ice, centrifuged at 14,500 rpm for 10 min at 4°C, and the supernatants recovered. The protein concentration was measured using Bradford Protein Assay kits (Bio-Rad), and 30 µg of each protein was electrophoresed using 10% SDS-PAGE. The proteins were transferred to a PVDF membrane (Immobilon-P, Merck), blocked with 5% skim milk, and incubated with anti-SMAP1 and AGFG2 antibodies. The peroxidase-conjugated secondary antibody was incubated and reacted using ECL kits (Cytiva).The signal was detected using ChemiDoc XRS+ (Bio-Rad). The membrane was stripped using stripping buffer (strong, NAKALAI) for detecting the β-actin signal as a loading control.

To investigate vWF processing, 50×10^4^ HUVECs were electroporated and seeded on a six-well plate. After 24 h, the medium was changed and the cells incubated for 48 h. The cells were scraped in 100 µl of lysis buffer, lysed by being drawn into and expelled from a 23 G needle five times, and kept on ice for 30 min. After centrifugation, the supernatant was taken and subjected to western blotting.

### Generation of SMAP1 KO cell line

SMAP1 guide RNA was designed using the online design tool CRISPR direct (Naito et al., 2015). The selected gRNA targeted exon 2 of human SMAP1. The gRNA sequence was ordered as a pair of oligonucleotide 5′-CACCGATATTCCAGGAAGCCCATCG-3′, and 5′-AAACCGATGGGCTTCCTGGAATATC-3′. The oligonucleotides were annealed and inserted into the BbsI site of pSpCas9(BB)-2A-Puro(PX459)V2.0 (Addgene, Watertown, MA, USA), which has Cas9 and gRNA expression vectors. HEK293 cells were transfected with SMAP1-targeted PX459 vector using Lipofectamine 3000, incubated for 2 days, then 1 µg/ml Puromycin (Thermo Fisher Scientific) was added. After 9 days, the cells were diluted to 1 cell/100 µl medium, and 100 μl plated in each well of a 96-well plate. The cells were grown, and the genome was purified using QIAquick Gel Extraction kits (Qiagen, Hilden, Germany). A fragment of around 300 bp, including the SMAP1 PAM site, was amplified by the primer 5′-ACTGCCGCCAGTACCATTTG-3′, and 5′-TGGTAACGTCATGTTTACCTGTATC-3′, and sequenced by the nested primer 5′-TTGAGCATGTGACTTCTGTAAGC-3′. The SMAP1KO A2 cell line has a deletion of 14 bp after the PAM sequence, and the SMAP1KO C4 cell line has a T insertion in one allele, and an 11 bp deletion in a second allele. Both SMAP1KO cell lines were subjected to western blotting (Fig. 2E).

### ELISA

20×10^4^ HUVECs were electroporated with control, SMAP1 or AGFG2 siRNAs, and seeded on 24-well plate coated with 0.3 mg/ml collagen type IV. After 16–20 h, the medium was changed. Forty-eight hours after the medium change, the cells were washed twice with 0.1% BSA/Hank's Balanced Salt Solution (HBSS, Merck) and incubated for 1 h with 0.1% BSA/HBSS. The cells were stimulated with either DMSO or 100 ng/ml PMA in 500 µl of 0.1% BSA/HBSS for 30 min. For histamine treatment, either water or 100 μM histamine (Merck, H7125) was added instead of DMSO or PMA. The medium was collected, centrifuged at 1000 rpm for 10 min at 4°C and stored at −30°C. The cells were washed with PBS twice, lysed with 500 µl of ELISA lysis buffer (0.5% Triton X-100, 1 mM EDTA/PBS) and stored at −30°C. Anti-vWF antibody was diluted in carbonate buffer (pH 9.6) at 1:400 for coating 96-well plates for 3 h at 37°C. The coated plate was washed three times with washing buffer (0.1% Triton x-100/PBS), incubated with blocking buffer (0.1% Triton X-100, 0.2% gelatin/PBS) for 30 min at RT, and incubated with 180 µl of the sample and 20 µl of blocking buffer (total 200 µl) overnight at 4°C. The plate was washed three times with washing buffer, and incubated with anti-vWF-HRP (P0226, Agilent Technologies Inc., Santa Clara, CA, USA) diluted 1:4000 in blocking buffer for 90 min at RT, then washed five times with blocking buffer. One tablet of 10 mg of O-Phenylenediamine dihydrochloride (Merck) was dissolved in 25 ml of citrate buffer, 200 µl added to each well, and the reaction started by adding 10 µl of 30% H_2_O_2_. After 3–4 min, the reaction was stopped with 50 µl of 2 M H_2_SO_4_. The absorbance at 492 nm was measured using a plate reader (Powerscan HT, SD pharma, Osaka and Tokyo, Japan). The percentage of secretion was calculated by dividing the amount of vWF in the medium by the total amount of vWF in the medium and the lysate.

### TEM

For TEM, 25×10^4^ HUVECs were electroporated and plated on gelatin-coated coverslips. The medium was changed after 24 h. Seventy-two hours post-transfection, the cells were fixed in 1% glutaraldehyde/4% PFA for more than 7 days. The cells were quenched with 50 mM NH_4_Cl. The cells were treated with 1% osmium tetroxide, washed with PBS and 1% tannic acid for 30 min, dehydrated with ethanol, and embedded in Epok. Sections 100 nm thick were stained with U and Pb, and observed using electron microscopy (JEM2100, JEOL, Ltd, Tokyo, Japan).

### vWF multimer analysis

For multimer analysis, 15–20×10^4^ HUVECs were electroporated and plated on collagen-coated 24-well plates. After 16–20 h, the medium was changed, and the cells incubated for another 48 h. Seventy-two hours post-transfection, the cells were pretreated with 0.1% BSA/HBSS for 1 h, then incubated with 0.1% BSA/HBSS including either DMSO or 100 ng/ml PMA for 30 min. The medium was collected, centrifuged at 1000 rpm for 10 min at 4°C and stored at −30°C. The experiments were performed in triplicate, and the 20 μl of media from all samples were mixed with sample buffer [30%(v/v) glycerol, 50 mM Tris-HCl, 10% SDS, 250 mM DTT, 10 mM EDTA, 0.1%(w/v) BPB, pH 6.8] without DTT and electrophoresed on the same agarose gel. Normal human plasma (NP, Siemens Healthineers, Erlangen, Germany) was also electrophoresed, as a control. The gel was blotted on membrane and detected using anti-vWF primary antibody (Agilent DAKO, Carpinteria, CA, USA) and IRDye 800CW-labeled anti-rabbit IgG secondary antibody (LI-COR Bioscience, Lincoln, NE, USA) as previously described (Naito et al., 2016). The near-infrared fluorescence signal was detected using an Odyssey CLx Imaging System (LI-COR Biosciences), and the intensity of each band was quantified using ImageJ (Schneider et al., 2012). The smallest five bands from smallest were considered to be ‘low-molecular weight (LMW)-vWF’, bands 6 to 10 were ‘middle molecular weight (MMW)-vWF’, and those more than 11 were ‘high-molecular weight (HMW)-vWF’. The intensity of HMW-vWF was divided by the total vWF intensity in the same lane to produce the large-multimer ratio. Then the LMI was calculated as the ratio of NP to the large-multimer ratio (Tamura et al., 2015).

## Supplementary Material

Supplementary information
