# Supplementary material for: Arf GTPase-activating proteins SMAP1 and AGFG2 regulate the size of Weibel-Palade bodies and exocytosis of von Willebrand factor
Source: Biol Open. 2021 Sep 1;10(9):bio058789. doi: 10.1242/bio.058789 (PMC8430232; doi:10.1242/bio.058789)
Supplement: Supplementary information [file biolopen-10-058789-s1.pdf]

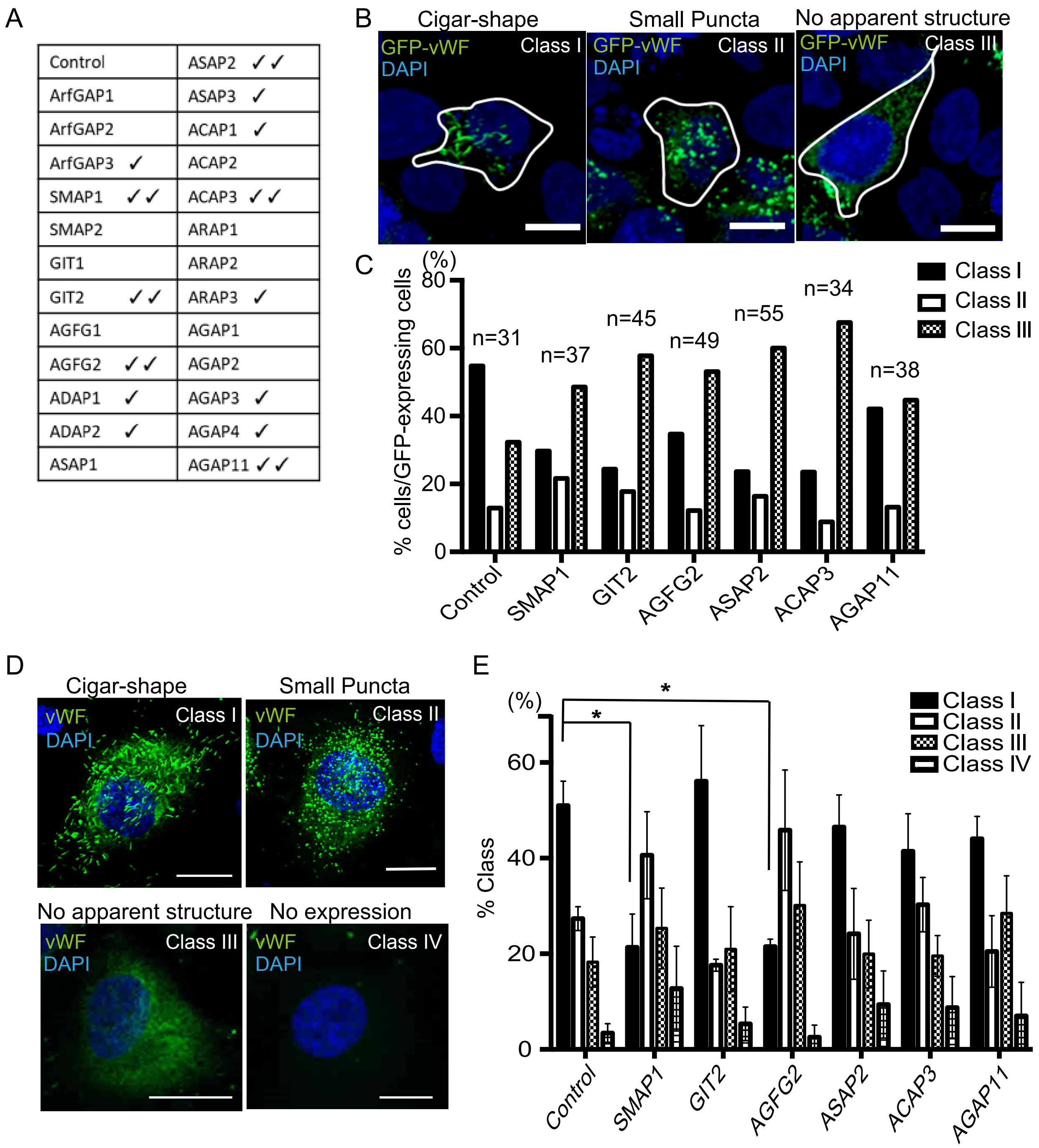

Control	ASAP2 ✓✓
ArfGAP1	ASAP3 ✓
ArfGAP2	ACAP1 ✓
ArfGAP3 ✓	ACAP2
SMAP1 ✓✓	ACAP3 ✓✓
SMAP2	ARAP1
GIT1	ARAP2
GIT2 ✓✓	ARAP3 ✓
AGFG1	AGAP1
AGFG2 ✓✓	AGAP2
ADAP1 ✓	AGAP3 ✓
ADAP2 ✓	AGAP4 ✓
ASAP1	AGAP11 ✓✓

Fig. S1. The siRNA screening by HEK293 cells and HUVECs. A. HEK293 cells were transfected with GFP-vWF and ArfGAP siRNAs and observed by confocal microscope. The cells that have apparently pseudo-WPBs were eliminated. Fourteen samples were kept for second screening, The same analysis was performed and 6 sample remained. The check mark was for ArfGAPs that were subjected for next analysis. **B.** HEK293 cells were transfected with GFP-vWF (green) and the nucleus was stained with DAPI (blue). The images were captured using a confocal microscope, and the cells were classified into three classes. Scale bar, 10 µm. **C.** HEK293 cells transfected with the six siRNAs were classified into three classes and the percentage of each class in the GFP-expressing cells were calculated. More than 30 cells were analyzed, as described. **D.** HUVECs were stained with anti-vWF and DAPI. The cells were classified according to WPB structure. Scale bar, 15 µm. **E.** HUVECs transfected with the six siRNAs were classified into four classes, and the percentage of each class in all cells was calculated. More than 40 cells were classified, and the experiment was repeated three times (n = 3). One-way ANOVA followed by Dunnett's multiple comparison tests were performed to compare each class with control cells. In SMAP1 and AGFG2 siRNAs-transfected cells, the number of cells in Class I was decreased. * $p < 0.05$, error bar; standard error of means (SEM).

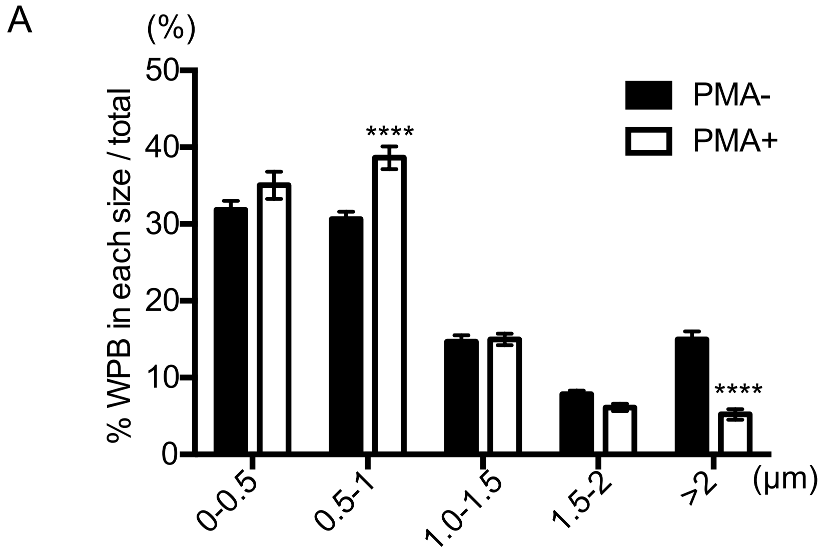

Fig. S2. WPB size in HUVECs with or without PMA. HUVECs were plated in coverslips, and after 20 hr, the medium was changed. Forty-eight hours after the medium change, the cells were serum starved for 1 hr and treated by 100 ng/ml PMA for 30 min. The cells were fixed and stained for vWF. WPB size was quantified for 10 cells per experiment, and the experiment was repeated three times (n = 30 for both). Two-way ANOVA with Sidak's multiple comparison tests were performed with or without PMA. WPBs >2 μm was markedly decreased by PMA treatment. **** $p < 0.0001$.

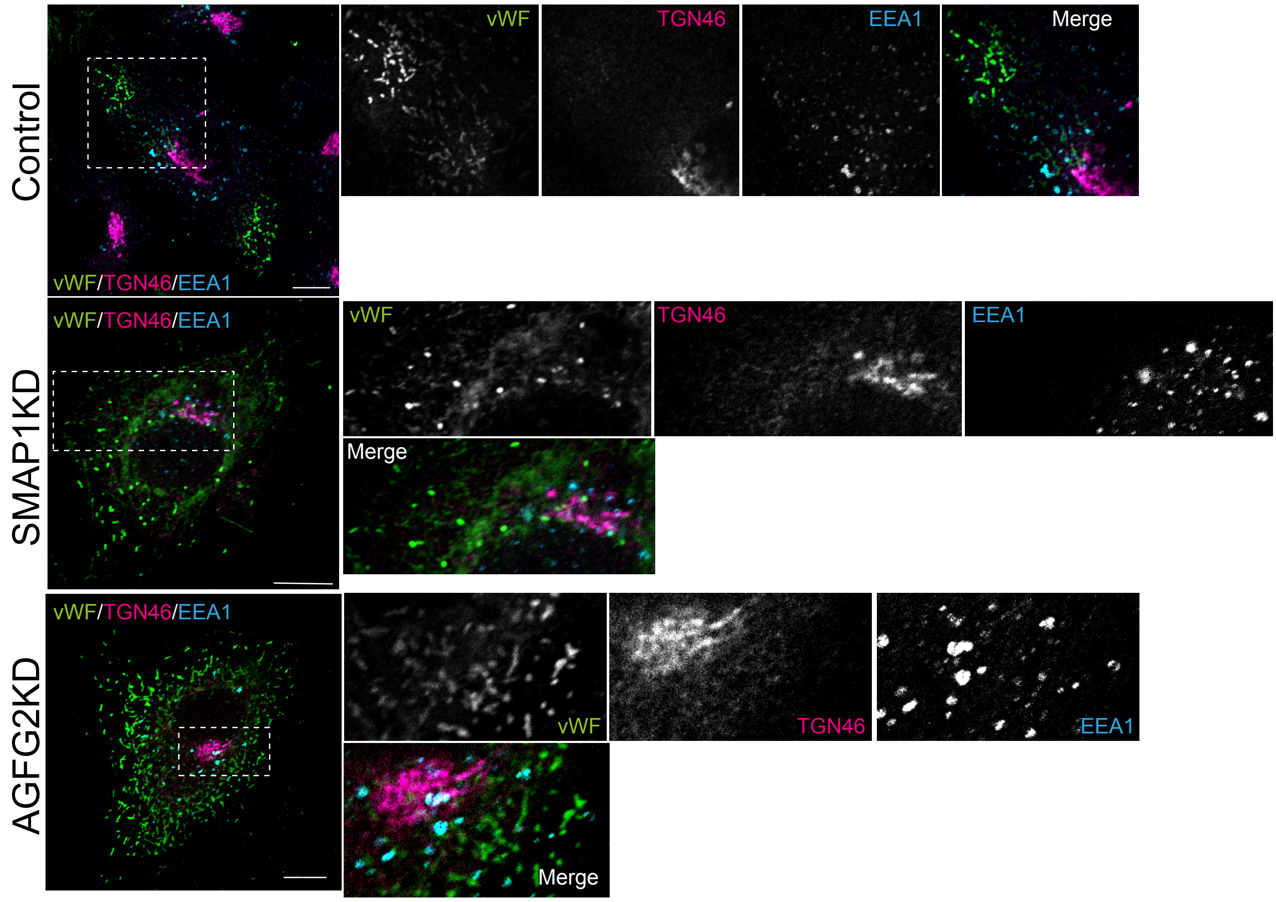

Fig. S3. The triple staining of vWF, EEA1, and TGN46 in control, SMAP1KD and AGFG2KD cells. HUVECs were electroporated with siRNAs as described, incubated for 72 hr, and stained for vWF (green), TGN46 (magenta) and EEA1 (Cyan). The area indicated by dashed boxes was enlarged. vWF did not colocalize with EEA1, and TGN architecture labeled by TGN46 was not largely altered in SMAP1KD and AGFG2KD cells. Scale bar 10 μm.
